# Input Pattern Classification Based on the Markov Property of the IMBT with Related Equations and Contingency Tables

**DOI:** 10.3390/e22020245

**Published:** 2020-02-21

**Authors:** István Finta, Sándor Szénási, Lóránt Farkas

**Affiliations:** 1 Nokia, Bell Labs, Bókay street 36–42, H-1083 Budapest, Hungary; lorant.farkas@nokia-bell-labs.com; 2 Department of Informatics, J. Selye University, Bratislavská cesta 3322, SK-94501 Komárno, Slovakia; szenasis@ujs.sk

**Keywords:** data structure, balanced binary tree, bipartite graph, Bernstein theorem, Fibonacci sequence, Markov property, state space, contingency table

## Abstract

In this contribution, we provide a detailed analysis of the search operation for the Interval Merging Binary Tree (IMBT), an efficient data structure proposed earlier to handle typical anomalies in the transmission of data packets. A framework is provided to decide under which conditions IMBT outperforms other data structures typically used in the field, as a function of the statistical characteristics of the commonly occurring anomalies in the arrival of data packets. We use in the modeling Bernstein theorem, Markov property, Fibonacci sequences, bipartite multi-graphs, and contingency tables.

## 1. Introduction

In large-scale or distributed systems, different types of data are generated locally, in the computer nodes composing the system. However, if the generated data also has to be handled or stored in a remote location, then the data has to be broken into packets and transmit over the network towards their destination. Since neither the transmission networks themselves, nor the consisting devices are perfect, some of the packets may arrive out-of-order, while others may be lost permanently. Additionally, packets that are mistakenly considered to be permanently lost during the transmission are sent several times. This may result in duplication on the receiving side.

Applications need to be prepared to handle out-of-order delivery, packet duplication, and packet loss, to an extent depending on the application specifics. A video application might neglect them overall, while a banking application might have very strict requirements to mitigate them. The efficiency of handling these phenomena depends on the underlying data structures used for the administration of packet arrival.

Binary search trees [1] are widely used in several fields of computer science. Their behavior is well studied, and under certain conditions, e.g., when assuming that the trees are balanced, the average cost of a search operation as a function of the number of nodes can be easily estimated. Nevertheless, traditional BSTs store all the keys. However, there are situations when it is more important to decide whether a key is already in the data structure or not, such as in the case of an Extract Transform Load (ETL) application. Hash tables [1] represent a solution in which the average time complexity of search operation is O(1). However, in so called long running systems, the requirement to keep all the keys for a long period to ensure duplication-free operation, translates to proportionally high storage space requirement, like CHORD [2].

To detect packet loss and duplication, a special, tree-like data structure was proposed, the Interval Merging Binary Tree (IMBT) [3], appropriate for cases when the keys are sequentially ordered by design, or there is a possibility to map the keys onto the natural numbers.

In [4], we performed an analysis of IMBT in a scenario where packets arrive out-of-order according to arrival processes most frequently experienced in practical cases. We applied this data structure over a stream processing framework, like Storm [5].

As part of an additional analysis [6] the full state-space of IMBT has also been determined. In‘contrast to a data structure like AVL balanced BST [7] or Red Black balanced tree [8], in the case of IMBT, it is not possible to determine the average complexity of the search operation as the exclusive function of the already stored keys because states are more than one-dimensional, even if the tree is balanced. Therefore, for each number of stored keys, *N*, we had to give an estimate of the possible number of different average time complexities. The state-space is an exponential function of *N*. An additional outcome of the analysis revealed that the contingency table [9] is, by design, suitable to classify different statistical input key distributions [10,11]. Therefore by the association of a particular occurrence of the contingency table with *N* it is possible to determine the average time complexity of the operation in question.

The purpose of this contribution is to give a classification of the average cost of search operation based on the Markov property [12,13] of the process represented by the number of nodes in the tree which depends on the pattern of packet losses/duplications at the input. Since the observed random variables are dependent, the weak dependent related Bernstein-criterion/theorem [14,15] is also considered during the examinations. We use contingency tables to visualize these evolutions.

The paper is divided as follows. In Section 2 we briefly summarize the data processing environment and the main characteristics of IMBT, then we introduce the role of contingency tables. Then in Section 3 we discuss the two main cases, first when the gaps in the originally continuous string of keys are permanent ones, and second when the loss of keys are temporary ones and gaps are gradually filled. In Section 3.1 we introduce the case of permanent gaps and in Section 3.2 we describe the case of temporary gaps. Section 4 contains proofs and deductions related to the formulated theorems. In Section 5 the theoretical results will be compared to existing data structures. Additionally, in this section we introduce the outcome of some selected simulations, which confirm the theories.

## 2. Interval Merging Binary Tree and Contingency Table

### 2.1. The Data Processing Environment and the Motivation Behind IMBT

**Figure 1:**
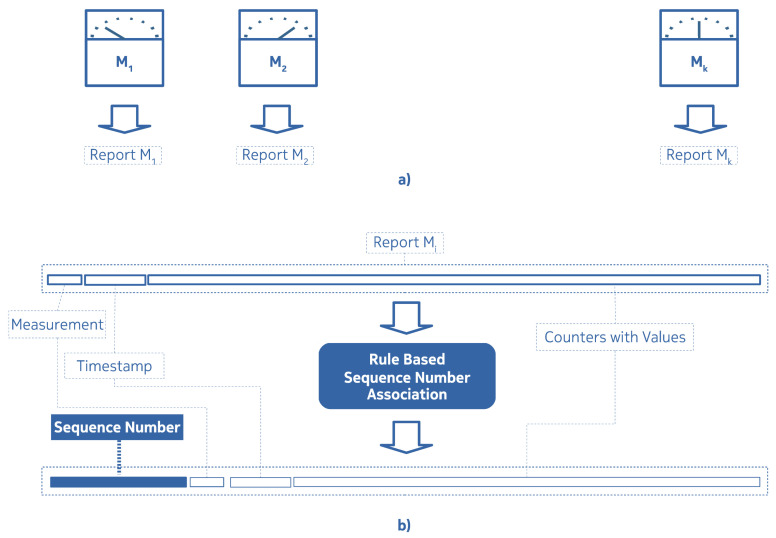
(**a**) Meters with periodically emitted measurement reports. (**b**) Rule-based sequence number association.

Consider an environment where the group of distributed measuring instruments, say that *k* instances ($M_{1},M_{2},$…$M_{k}$), emit their measurement reports $R_{Mi}$ periodically, as seen in Figure 1a. Each instrument has its own unique identity. Our goal is to collect, normalize, and transport these measurement reports, along with guaranteed duplication filtering and high-speed (near real-time) processing. To achieve this, first, we apply the following mapping rule: During the first period the $R_{M1}$ report from $M_{1}$ is associated with number 1 ($R_{M1}⟼1$), and subsequently $R_{M2}⟼2,\;$…$\;,R_{Mk}⟼k$. Supposing that the time of report generation is part of the unique identity, like in case of attribute-based naming (or one can easily extract it from the report), from the second period we apply the mapping $R_{M1}⟼\left(k+1\right),\;R_{M2}⟼\left(k+2\right),\;$…$\;,R_{Mk}⟼\left(k+k\right)$, from the third period: $R_{M1}⟼\left(2k+1\right),\;R_{M2}⟼\left(2k+2\right),\;$…$\;,R_{Mk}⟼\left(2k+k\right)$, etc., based exclusively on the unique identity and the time of the report generation as shown in Figure 1b.

Thermometers of the national weather service might be an example of endpoints for such a system.

In the following, we consider the case when the reporting period might differ instrument group by instrument group. Let us denote by $rp_{1},rp_{2},$…$,rp_{l}$ the different reporting periods in increasing order (longer period is marked by higher index). For the sake of simplicity, suppose that all the longer periods are integer multiples of all the shorter ones. That is, $rp_{2}=a\times rp_{1}$, $rp_{3}=b\times rp_{1}$ and $rp_{3}=c\times rp_{2}$, such that a,b,c∈N. Since $b\times rp_{1}=c\times rp_{2}\Rightarrow b=ac$. In this case, it is also easy to create a set of substitution rules, aided by the fact that the measurements can be linearized. Under linearization, here we mean such an operation where during the determination of the assignable intervals (contiguous sequence of integer numbers) of measurements with a longer period, we recursively consider that ranges of sequential numbers, whose ranges were assigned before to the shorter periods measurements.

One can think about the linearization as the dimension reduction of two-dimensional data with Hilbert space-filling curve [16].

Examples for such a mixed reporting environments might be the IoT (Internet of Things) reporting entities of modern agro-meteorology systems, where different measurement features require different measurement periods; or a physical experiment, where different measurements, observing the same event, depend on the feasible/available granularity.

**Figure 2:**
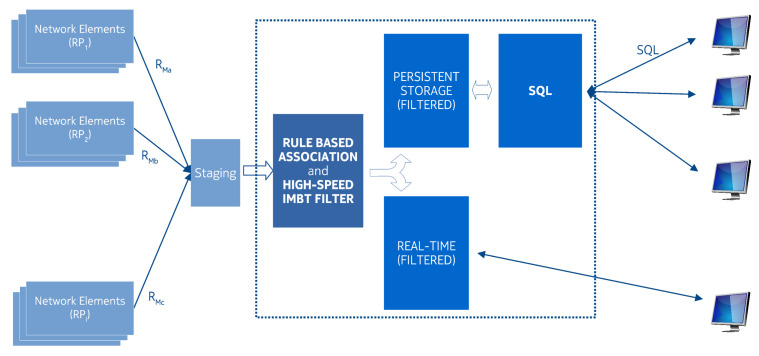
Mobile network: an example for mixed measurement periods.

The mobile networks can also be considered such a system, in which mixed/heterogeneous measurement periods exist: In the case of a mobile network, ten thousand network elements continuously measure several parameters (signal strength, dropped packages, dropped connections, location updates, etc.) with a different emitting period. In an extensive mobile network, the geographical distribution is also an important factor. Due to its distributed nature, there is no guarantee that during the raw data collection all the raw data will travel in the same order on the same path towards the central repository, even from the same source. Therefore, out-of-order arrival or temporary/final loss and retransmission also might take place. To be able to process the results near real-time, the application of a fast filtering method is essential. Due to the near real-time criterion, a traditional database is out of the question. By the application of a constant time hash table, the filtering speed could be satisfied, however, considering the enormous amount of data it can become a bottleneck from a memory consumption point of view. To reduce the memory consumption and increase the throughput of a processing system that operates in the above described/characterized environment, the IMBT is introduced, as seen in Figure 2.

In an idealized environment, when all the reports eventually arrive, as we shall see, the height of the tree is stabilized around an expected result. Therefore, the time complexity of the average search operation can be considered to be constant, that is, O(1).

However, the measuring environments are usually far from ideal. Therefore, for instance, due to the failure of a network element, the measurement reports might not have been generated at all, or the reports are lost eventually over the transmission, and final gaps appear in an ideally continuous interval, which causes a variously increasing average cost of the search operations.

### 2.2. Interval Merging Binary Tree

**Figure 3:**
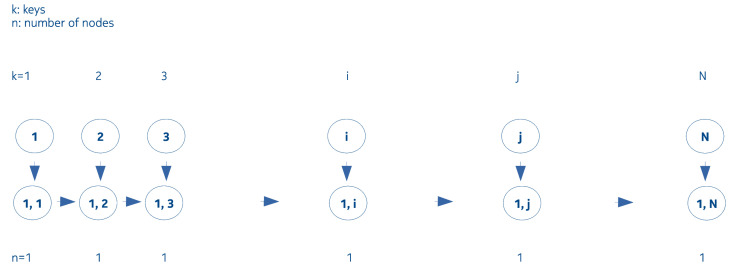
Interval Merging Binary Tree (IMBT) number of keys increasing and the interval evolving while the number of nodes is constant.

We denote by interval a sequence of contiguous integer numbers, where the numbers represent the keys, ordinal numbers counting a sequence of data packets received by a host in the network. Interval Merging Binary Tree is such a binary tree, in which, nodes are composed of nonoverlapping intervals that are not neighbors of each other. If we are only interested in if a certain packet arrived or not, it is enough to store only the extremes of the intervals instead of storing all the keys, as seen in Figure 3.

From the perspective of storage requirements, IMBT outperforms other data structures, storing all the keys if the average number of keys covered by the nodes of the tree is higher than 2. An additional data structure is required if we want to make an operation on the values associated to the individual keys, once it is decided, based on IMBT, whether a packet has arrived already, because IMBT reduces the space of keys to a space of intervals.

To count the possible arrangements of intervals we need to assess the number of distinct ways a number *N* of nodes can be partitioned into the sum of integers smaller than *N*. In the field of integer partitioning [17] the Hardy–Ramanujan number [18] represents an upper estimate for that:

(1) $$\lim_{N\to \infty }p\left(N\right)\approx \frac{1}{4N\sqrt{3}}e^{\pi \sqrt{2N/3}}$$

Now let us consider the fixed number of different keys *N*. Assuming that all the keys might be equally searched for, if *N* was stored in a traditional balanced binary search tree, then the average cost of a search operation can be expressed by the following formula:

(2) $$A\left(N\right)=log_{2}\left(N\right)$$

However, from Equation (1) it is clear that, by knowing only the already stored keys (that is *N*), we can get p(N) number of different combination of nodes, regarding interval lengths. We have not yet taken into account the effect of balancing either. Let us consider a simple example in which N=4 and let us denote the individual keys by $k_{1},k_{2},k_{3},k_{4}$. Based on the possible number of neighbors (neighborship being equivalent to the fact that the second packet is the immediate next packet to the first one), the following scenarios can be distinguished:

- None of the keys are neighbors of each other,
- Two of them are neighbors and the other two are not,
- Two of them are neighbors and the remaining two are neighbors as well,
- Three of them are neighbors and one is not,
- All the keys are neighbors of each other.

**Figure 4:**
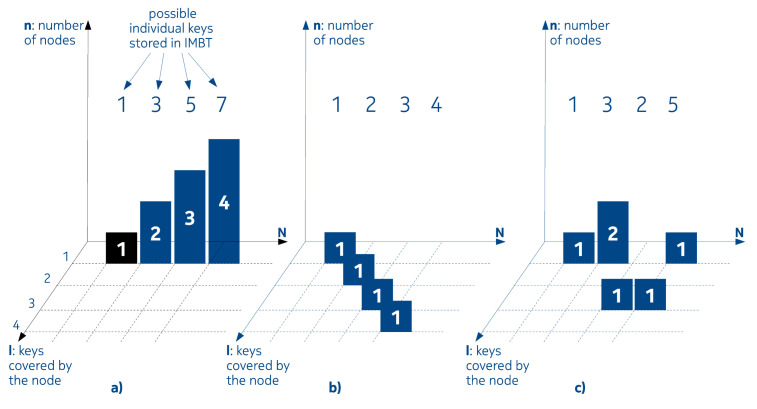
IMBT examples of evolving types for N=1…4. (**a**) No neighbors, (**b**) all keys are neighbors, (**c**) three keys are neighbors, one key is not.

We can see that these scenarios actually translate to the integer partitions of *N* when N=4: N=1+1+1+1, N=2+1+1, N=2+2, N=3+1N=4; that is p(N=4)=5 in this case. Three out of five scenarios are shown in Figure 4. Figure 4a corresponds to $k_{1}=1$, $k_{2}=3$, $k_{3}=5$, $k_{4}=7$. Figure 4b corresponds to $k_{1}=1$, $k_{2}=2$, $k_{3}=3$, $k_{4}=4$; while Figure 4c corresponds to $k_{1}=1$, $k_{2}=3$, $k_{3}=2$, $k_{4}=5$ arrival patterns. *N* represents discrete time of arrival of the packets. The remaining axes display the interval length of each node at a given time instant, represented by *N*.

In [6] we have proven that these arrangements with or without balancing can be mapped onto the rows and columns of a contingency table in the following way.

### 2.3. The Role of Contingency Tables on the Analysis of IMBT

Let us take a snapshot of IMBT and sort the intervals according to their length in monotone increasing order. Let *n* be the number of nodes in the IMBT. Let us denote by $l_{i}\in L$ the length of the intervals belonging to a node $n_{i}$ from the IMBT, where *L* is a multi-set. We can denote by *j* the number of distinct interval lengths. To all *j* we can assign a number d(j) that is the number of intervals with the same length associated to *j*. It is necessarily true that j≤n. These numbers d(j) are located in the header of the contingency table, that is, on the top of each column.

Now let us sort the intervals according to the distance of the nodes associated to them from the root node in a monotonic increasing order. Let us denote by $s_{i}\in S$ the number of comparisons required to reach the left hand value of an arbitrary node $n_{i}$, where *S* is a multi-set. The previously introduced $s_{i}$ is the distance. Then let us create as many $w_{i},\;i\in 1$…*k* for as many distinct distances there are. Finally let us assign to all $w_{i}$ a number $d(w_{i})$ meaning the number of intervals/nodes with the same distance from the root node. It is necessarily true that k≤n. These $d(w_{i})$ numbers are located in the right side of the contingency table, that is in the end of each row.

**Figure 5:**
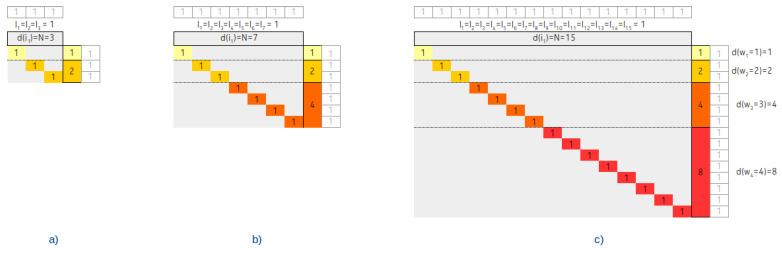
The balanced binary search tree related contingency tables. The (**a**–**c**) cases belong to the three different N values: 3, 7, and 15.

Two examples are presented in the following. First we represent a traditional, completely balanced BST in a contingency table, Figure 5.

**Figure 6:**
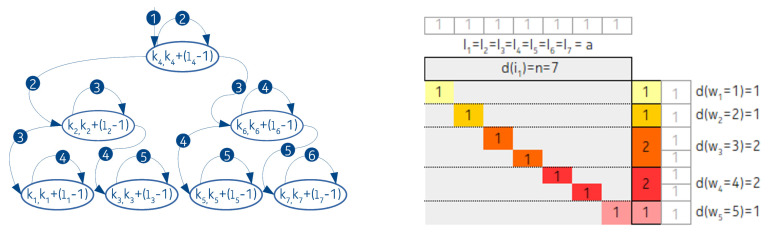
IMBT with seven nodes and the related contingency table. The arrows and the filled circles are marking the order and number of comparisons during the search operations.

In the second example, we show an IMBT and the related contingency table. For the sake of simplicity, suppose that there is a completely balanced IMBT with seven nodes, and the lengths of all the intervals are the same. That is, with the above notation: $l_{1}=l_{2}=l_{3}=l_{4}=l_{5}=l_{6}=l_{7}=a$. See Figure 6.

As can be seen in the figure, due to the equal length intervals, there is only one *j* and the number of intervals with the same length associated to it is d(j)=7. Of course, as we shall see, there are many arrangements where the interval lengths are different.

We can recognize two deviations between the contingency tables associated to BSTs and IMBTs. First, in the header of the contingency table belonging to the traditional BST, N=n number of keys appear. However in case of IMBT, the number of nodes *n* is present, since this number is not necessarily equivalent with the number of keys *N* covered by the tree so far. The second deviation is that although both trees are balanced, the number of nodes with the same distance from the root in case of BST follows the series of powers of two, but in case of IMBT it is a combination of a Fibonacci sequence and an additional term, as shown in [6].

## 3. Arrangements Related Conditions, Theorems, and Equations

By now we know a model in which we can count the average cost of search operation by simple multiplication of the corresponding values in the contingency table.

In the following, we will define some metrics and through these we will examine the evaluation of the contingency table.

Let us denote by *N* the number of keys stored in the IMBT so far just like above. However, unlike above, to be able to examine the evolution of the number of nodes in the tree, we introduce a random variable, $V_{i}$, which is the instantaneous number of vertices (nodes previously) in the tree at time instant *i*, where i∈1⋯N. It is obvious from the definition that $V_{1}=1$ and $V_{i}$ can be mapped to states in a stochastic matrix. The distribution of the lengths of the intervals is affected by the homogeneity and the finite/infinite nature of the stochastic matrix.

We define the series of instantaneous average interval lengths by the following formula:

(3) $$\bar{L_{i}}=\frac{l_{1}^{i}+l_{2}^{i}+\cdots +l_{V_{i}}^{i}}{V_{i}},$$

where *i* is the time instant (i∈1⋯N) and $l_{k}^{i}$ is the length of the interval stored by node *k* at time instant i. $\bar{L_{i}}$ is a random variable as well.

We assume that for the series of $\bar{L_{i}}$ the following constraints are true:

- There is an expected value *a*, to which the individual random variables, $a_{i}$, stochastically converge to as *N* tends to infinity, where $a=\lim_{N\to \infty }(a_{1}+a_{2}+$…$+a_{N})/N$.
- There is a $c=(\sigma_{1}^{2}+\sigma_{2}^{2}+$…$+\sigma_{N}^{2})/n$ independently from *N*, where $\sigma_{i}$ is the standard deviation of the interval lengths at time instant *i*.
- There is a non-negative function r(x) for which r(0)=1,limN→∞(r(1)+r(2)+…+r(n))/n=0, and additionally $|corr\left(\bar{L_{i}},\bar{L_{i}}\right)\left|\leq r(|i-j|\right)$, i,j≥1.

The conditions mentioned above together constitute the Bernstein-theorem [14]. According to the theorem, if the three constraints are simultaneously met, then the weak law of large numbers is true.

Using the Bernstein theorem as a starting point, we can identify two types of completely different input pattern classes, for which the behavior of contingency tables is examined and the cost of average search operation is determined:

- In the first case only a nonrecurring, transient, infinite state stochastic matrix can be composed based on the associated states $V_{i}$. Additionally we assume that the Bernstein-theorem is true for the series of $\bar{L_{i}}$,
- In the second case, we assume that based on the state $V_{i}$, it is possible to create a stochastic matrix which has a finite state-space, is aperiodic, irreducible (that is ergodic), and recurrent.

The satisfaction of the first criterion implies that the average interval length is upper bounded. As a result, the variance of the interval lengths is also upper bounded, therefore, the scenario in which we have a composition of a large interval with continuously increasing length with increasing number of small ones, is not valid. Rather, as *N* increases, there will be an increasing number of gaps between the intervals, associated with permanently missing keys, that is, keys where the probability of arrival of the associated packet converges to zero. Both from the previous fact and from Equation (3) it is obvious that $V_{i}$ is proportionally increasing as well.

The satisfaction of the second criterion implies the presence of temporary gaps only: in spite of the increasing *N* the finite number of nodes implies that the instantaneous average interval length is increasing, that is the number of gaps is upper bounded.

### 3.1. Permanent Gaps

In the case of permanent gaps, the mean of the average interval length is a constant value, *a*. We do not exclude the possibility of having temporary gaps, due to the out-of-order arrival of packets. As a result, the header $d(i_{i})$ in the associated contingency table will follow a kind of distribution. Taking into account the effect of temporary gaps in the analysis would make the analysis more complex, but their effect is minor, therefore they will be discarded in the subsequent.

#### 3.1.1. Linked List Arrangement

In this realization, our additional assumption against the keys is that there is a smallest one.

**Figure 7:**
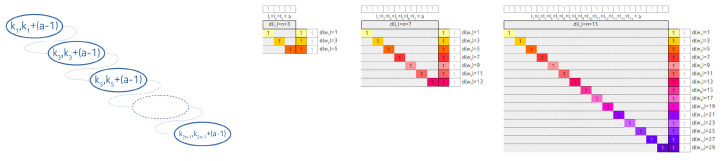
Linked list degenerated IMBT and three associated contingency tables.

The tree degenerated into a linked list and three associated contingency tables with $V_{i}=n=3$, $V_{i}=n=7$ and $V_{i}=n=15$ are shown in Figure 7.

**Theorem** **1.** *In the case when there is no shuffling and no balancing at all, the tree degenerates to a linked list and*

(4) $$A\left(N,a\right)=\frac{N}{a}+\frac{a-1}{a}.$$

From the contingency table, it is clearly visible that the $w_{i}$ follows the sequence of odd numbers and $d(w_{i})$ remains constant.

#### 3.1.2. Completely Balanced Arrangement

**Figure 8:**
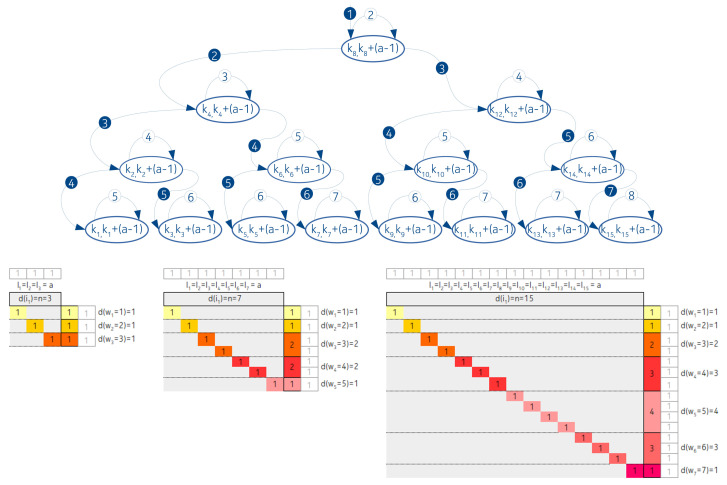
Completely balanced IMBT and three associated contingency tables.

We assume a completely balanced tree with the number of node power of 2, that is $n=2^{l}-1$. Three associated contingency tables with $V_{i}=n=3$, $V_{i}=n=7$ and $V_{i}=n=15$ are shown in Figure 8.

**Theorem** **2.** *With the above conditions, the average cost of the search operation can be expressed with the following formula:*

(5) $$A\left(N,a\right)=\frac{3}{2}log_{2}\left(\frac{N}{a}+1\right)+\frac{3a}{2N}log_{2}\left(\frac{N}{a}+1\right)-\frac{a+1}{a}.$$

As is visible from the figure and as we have proven in [6], the balancing has a typical fingerprint in the $d(w_{i})$ distribution: It follows the Fibonacci sequence until the middle of the rows on the way from the top rows to the bottom rows.

Supposing that N>>0, and a>0. Then we can apply the following simplification on Equation (5):

(6a) $$\begin{array}{cc} A\left(N,a\right)\approx \frac{3}{2}\log_{2}\left(\frac{N}{a}\right) & =\frac{3}{2}\log_{2}\left(N\right)-\frac{3}{2}\log_{2}\left(a\right) \end{array}$$

(6b) $$\begin{array}{c} =\log_{2}\left(N\right)+\frac{1}{2}\log_{2}\left(N\right)-\frac{3}{2}\log_{2}\left(a\right). \end{array}$$

That is, an IMBT with the given criteria will outperform a BST as long as the difference of the second and third term is negative, in other words, provided that:

(7) $$a>\sqrt[{3}]{N}$$

### 3.2. Temporary Gaps Only

Let us assume in the following cases that it is possible to compose from $V_{i}$ a finite state {1…p}, ergodic (aperiodic, positive recurrent), at least partially recurring stochastic matrix.

Let us denote by $M_{j}$ the mean recurrence time in state *j*. If $M_{j}$ is finite then state *j* is positive recurrent. The state *j* in which the expected return time is the smallest one, will represent the number of nodes in the IMBT as a steady-state value. Therefore we can substitute that state with value n=j.

Regarding the distribution of the interval lengths, which are necessarily increasing, we will distinguish two possible realizations.

- In the first realization we suppose that the increasing interval lengths are uniformly distributed to nodes, the number of which is fixed.
- In the second realization the distribution is not uniform.

#### 3.2.1. Linked List Like Arrangement

In this subsection, our additional assumption against the keys is that there is a smallest one, which we may always call first and as time goes by the probability that all the keys near the first key have already arrived is increasing.

Since the gaps are temporary ones, out-of-order arrival implies there are keys which arrive late, therefore temporary side-branches might appear over time, outside of the main branch. The length of these temporary branches depends on the statistics of the out-of-order arrival pattern. Subsequent side-branches are not taken into account because their effect is marginal.

**Theorem** **3.** *With the distribution characterized above, the data structure degenerates into a linked list. Suppose that the increasing lengths are uniformly distributed across the nodes. Then, due to the uniformly distributed increasing lengths, the average cost of the search operation does not depend on N:*

(8) A(N,a)=n.

Figure 7 accurately describes this scenario as well.

In the following, we suppose that the distribution of the lengths is not uniform, but node-heavy, meaning that every node contains a single key only, except one, which contains all the remaining N−n+1 keys. That heavy node can reside at the tail, in the middle, or at the root of the linked list degenerated tree.

**Theorem** **4.** *Then the average costs of search operations in case of node-heavy arrangements are the followings:*

(9a) $$\lim_{N\to \infty }A_{tail\;heavy}\left(N,a\right)=2n.$$

(9b) $$\lim_{N\to \infty }A_{middle\;heavy}\left(N,a\right)=n.$$

(9c) $$\lim_{N\to \infty }A_{root\;heavy}\left(N,a\right)=2.$$

**Figure 9:**
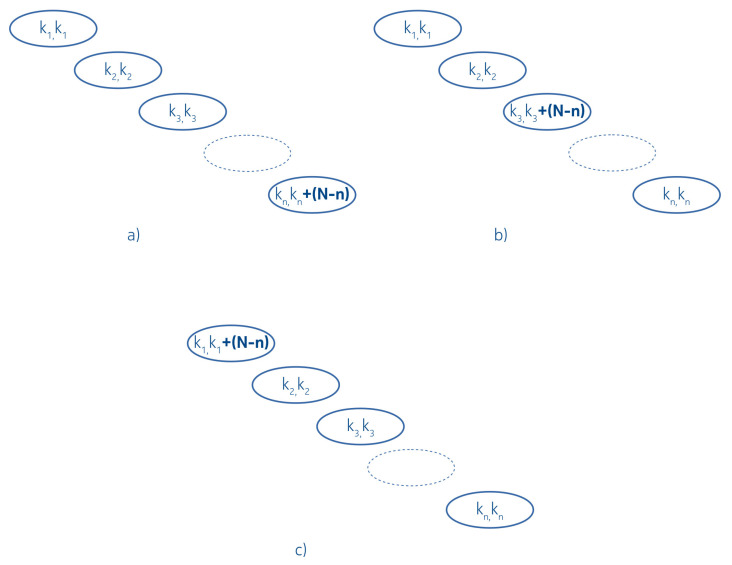
The linked list degenerated IMBT with heavy nodes. (**a**) is tail heavy, (**b**) is middle heavy and (**c**) is root heavy IMBT.

**Figure 10:**
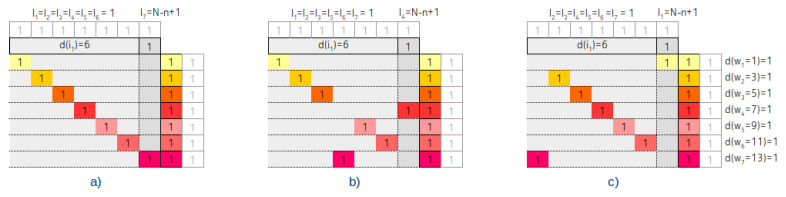
The associated contingency tables of linked list degenerated IMBT with heavy nodes. (**a**) is tail heavy, (**b**) is middle heavy and (**c**) is root heavy contingency table.

The related arrangements and contingency tables are shown at Figure 9 and Figure 10 respectively.

#### 3.2.2. Completely Balanced Arrangement

Two scenarios are considered:

- The increasing lengths of intervals are uniformly distributed across the tree,
- The distribution of lengths follows an exponential distribution.

The first case is fairly simple. Since the number of nodes is fixed with value *n*, it is easy to see the following.

**Theorem** **5.** *considering a completely balanced IMBT the average cost of search operation is such a constant, which is proportional with the logarithm of n. Based on Equation (6b), considering that a>>0 (since a increasing infinitely) we get:*

(10) A(N,a)≈C(n)−1.

From the formula it is visible that, just like in case of Theorem 3, the A(N,a) is independent from *N*.

The other case is a little bit trickier: it depends on the length distribution. Suppose that the nodes with smaller key values hold the longer intervals, while nodes with the highest key values hold the shorter intervals. Additionally, the rightmost interval is always one. Compensating this constraint without increasing the number of nodes the leftmost interval always absorbs the surplus.

We do not give the related formula and the associated deduction here, but consider the construction of a similar, however, "more realistic" arrangement in the next subsection.

#### 3.2.3. An Exception: Completely Balanced Arrangement, Temporary Gaps, Infinite Nodes, and Increasing Average

In this subsection, we introduce an arrangement, where none of the two criteria from Section 2 hold: the average length of the intervals is increasing, along with the increasing number of nodes. Our initial assumption is that the interval lengths are exponential according to power of two. Additionally the longest interval has the smallest left-hand key value, the second longest interval has the second smallest left-hand key value and so on. Moreover we suppose that the length of the shortest interval is always $2^{0}$.

Since we are examining asymptotic results we apply the simplification that only the right side distances will be taken into account for the determination of the full weight of IMBT. That is, if the length of an interval is $l_{i}\left(=2^{i}\right)$ then, with this approach we weight the right distances of a node by the full $l_{i}$, instead of $l_{i}-1\left(=2^{i}-1\right)$. However, it is easy to see that as N→∞ this difference becomes insignificant.

By considering a tree nodes arrangement and applying the above constraints we obtain interval lengths of $2^{0},2^{1},2^{2}$. As we stipulated before, the longest interval has the smallest left key value. Therefore, in a balanced IMBT $2^{2}$ interval has the distance from the root 3 comparisons (we take into account the right hand distances). The $2^{1}$ interval is the root node, therefore, we count with 2 comparisons. The 1 key interval is in the right side of the balanced IMBT, therefore to reach the majority of that keys requires 4 comparisons.

As we stated before, during the calculations we assumed that the following conditions are hold:

- Any key can be the subject of the search operation with equal probability,
- The key is already in the tree.

Therefore, during the determination of the average cost of search operation, the actually expected value of comparisons is calculated. According to our assumption the probability of we are looking for key *k* is 1/N, where k∈{1…N}. Since during the jth comparison several keys can be found the jth comparisons has to be weighted with the length of the intervals. According to the above mentioned the A(N) average cost is 1/N multiplied by the sum of weighted nodes, where a particular weight, which belongs to a single node is the multiplication of the distance and the length of the interval. The sum of weighted nodes, that is the total weight, is denoted by TW.

Let us assign the $s_{1},s_{2}$ and $s_{3}$ to the above numbers, respectively. That is, $s_{1}=3,s_{2}=2$ and $s_{3}=4$. The approximate value of the total weight of the tree is the following:

(11) $$TW=s_{1}2^{0}+s_{2}2^{1}+s_{3}2^{2}$$

Now by extending our examination to a n=7 nodes arrangement, the longest interval is $2^{6}$. The comparison weights depend on the lengths and the distances from the root, therefore in this case we obtain:

(12) $$\begin{array}{c} TW=\left(s_{1}+2\right)2^{0}+\left(s_{2}+2\right)2^{1}+\left(s_{3}+2\right)2^{2}+\left(s_{1}+1\right)2^{0+4}+\left(s_{2}+1\right)2^{1+4}+\\ \left(s_{3}+1\right)2^{2+4}+\left(s_{2}+0\right)2^{3} \end{array}$$

From the above two equations we can formulate the recursive extension/composition rule: Take the given expression which is valid for *n* nodes. To determine the 2n+1 nodes arrangement, first copy the whole formula and increase by 2 the multipliers of the powers. Then add the formula with the multipliers increased with one and powers increased by 2. According to modification 2, increase the multiplier values by 1. Additionally, add the corresponding base 2 value to the exponents. Finally add the missing new root member to the expression. To make it more understandable, here we give an extension of Equation (12).

(13) $$\begin{array}{cc} TW & =\left(s_{1}+2+2\right)2^{0}+\left(s_{2}+2+2\right)2^{1}+\left(s_{3}+2+2\right)2^{2}+\\ \; & +\left(s_{2}+0+2\right)2^{3}+\left(s_{1}+1+2\right)2^{0+4}+\left(s_{2}+1+2\right)2^{1+4}+\left(s_{3}+1+2\right)2^{2+4}+\\ \; & +\left(s_{1}+2+1\right)2^{0+8}+\left(s_{2}+2+1\right)2^{1+8}+\left(s_{3}+2+1\right)2^{2+8}+\\ \; & +\left(s_{2}+0+1\right)2^{3+8}+\left(s_{1}+1+1\right)2^{0+4+8}+\left(s_{2}+1+1\right)2^{1+4+8}+\left(s_{3}+1+1\right)2^{2+4+8}+\\ \; & +\left(s_{2}+0+0\right)2^{7}. \end{array}$$

**Figure 11:**
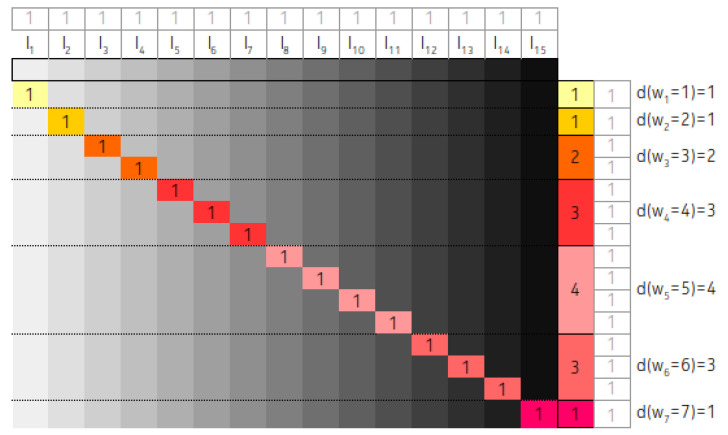
The contingency tables of IMBT where all the interval lengths are different.

This is such a recursive rule, that it affects both the multipliers and the exponents. The related contingency table is shown in Figure 11. In the figure, we indicated the number of steps that are required to achieve the interval opening keys, instead of the closing. That is, every weight associated values in the column are shifted by one.

Based on the figure and Equation (13) we can say that

(14) $$\begin{array}{cc} d\left(w_{1}\right)=1,\;where\;w_{1} & =(s_{2}+0+0)=2\\ d\left(w_{2}\right)=1,\;where\;w_{2} & =(s_{2}+0+1)=3\\ d\left(w_{3}\right)=2,\;where\;w_{3} & =\left(s_{2}+0+2\right)=\left(s_{2}+1+1\right)=4\\ d\left(w_{4}\right)=3,\;where\;w_{4} & =\left(s_{2}+1+2\right)=\left(s_{2}+2+1\right)=\left(s_{1}+1+1\right)=5 \end{array}$$

etc.

Since the average length of the intervals is strictly tied to *N*, the average cost of search operation is depending exclusively on *N*, that is

(15) $$A\left(N\right)=\frac{1}{N}\times TW_{N}.$$

## 4. Proofs and Deductions

In this section we give the proofs and deductions of the above results and theorems. During the deductions, it is assumed that any key has the same probability to be searched for. That is $P\left(the\;key\;we\;are\;looking\;for\;is\;k_{m}\right)=\frac{1}{N},where\;m\in (1$…N).

### 4.1. Permanent Gaps

#### 4.1.1. Linked List Arrangement

**Proof of Theorem** **1:** Every node contains two keys, since a node covers an interval and the keys represent the borders of that particular interval. Let us assume that a node in the tree covers *a* keys on average. Then we can denote by $k_{i}$ the starting key and by $k_{i}+\left(a-1\right)$ the ending key. Additionally, due to the non-overlapping feature of IMBT, it is also trivial that $\left(k_{i}+a-1\right)<k_{i+1}$.

First let us see the a=1 case. In this case in the linked-list degenerated data structure the starting and the ending keys are equal. As a consequence: if the key to be searched for is not equal with $k_{i}$ then the second comparison with the right value of that particular node is necessary but, due to $k_{i}==k_{i}+a-1$, the outcome of the comparison is always false.

Since the number of the nodes is n=N/a, and here a=1, therefore n=N. Based on this, we can write that the average cost of SEARCH is equal to the expected value:

(16) $$\begin{array}{cc} A(N) & =\frac{1}{N}\sum_{i=1}^{n}\left(2i-1\right)\\ \; & =\frac{1}{N}\left[2\frac{n(n+1)}{2}-n\right]=N. \end{array}$$

Now, we examine the a≥2 case. It is still valid that n=N/a. Analogous to the previous deduction we can write that the average cost of the SEARCH operation is:

(17) $$\begin{array}{cc} A(N,a) & =\frac{1}{N}\sum_{i=1}^{n}\left(2i-1\right)+2i\left(a-1\right)\\ \; & =\frac{1}{N}\left[n(n-1)-n+(a-1)n(n-1)\right]\\ \; & =\frac{N+a-1}{a}=\\ \; & =\frac{N}{a}+\frac{a-1}{a}. \end{array}$$

It is visible that substituting 1 into *a* we will return Equation (16).

An additional consequence is that as *a* grows the equation tends to:

(18) $$\frac{N}{a}+\frac{a-1}{a}\approx \frac{N}{a}+1.$$

With the deduction above, Theorem 1 is proved. □

#### 4.1.2. Completely Balanced Arrangement

From now on, the number of layers or levels is denoted by *l* (in contrast to the previous notation of lengths). The root node is the l=1.

**Proof of Theorem** **2:** Based on our notations we can write that:

(19) $$\begin{array}{cc} n= & \;\frac{N}{a},\\ l= & \;log_{2}\left(n+1\right)=log_{2}\left(\frac{N}{a}+1\right). \end{array}$$

During the deduction we will use the following identity:

(20) $$\sum_{i=1}^{n}i2^{i}=n\left(2^{n+1}-2\right)-\left(2^{n+1}-4\right)+\left(n-1\right)2.$$

Based on the relations (Equation (19)) we can define the layer level sums:

(21) $$2^{i-1}+2^{i}\left(a-1\right)+\left(i-1\right)2^{i-2}3+\left(i-1\right)2^{i-2}3\left(a-1\right),$$

where *i* means the $i^{th}$ level in the tree. However, this form is not suitable for equal transformations. Therefore, we split the formula into a fixed member which is the first node and the layer level members. Due to this split we will use an incremented *i* and the counter in the sum will last to l−1 instead of *l*:

(22) $$\begin{array}{cc} A(N,a)= & \frac{1}{N}[1+2\left(a-1\right)+\sum_{i}^{l-1}2^{i}+\left(a-1\right)2^{i+1}+3i2^{i-1}+3\left(a-1\right)i2^{i-1}]=\\ = & \frac{1}{N}[1+2\left(a-1\right)+\sum_{i}^{l-1}2a2^{i}-2^{i}+\frac{3a}{2}i2^{i}]=\\ = & \frac{1}{N}[1+a-a2^{l}-2^{l}+\frac{3a}{2}l2^{l}]. \end{array}$$

Since

(23) $$\begin{array}{cc} a-a2^{l}= & -a(2^{l}-1)=-N\\ 1-2^{l}= & -\frac{N}{a} \end{array}$$

we can write that

(24) $$\begin{array}{cc} A(N,a)= & \frac{1}{N}[\frac{3a}{2}l2^{l}-N-\frac{N}{a}]\\ = & \frac{3a}{2N}\left(\frac{N}{a}+1\right)log_{2}\left(\frac{N}{a}+1\right)-1-\frac{1}{a}\\ = & \frac{3}{2}log_{2}\left(\frac{N}{a}+1\right)+\frac{3a}{2N}log_{2}\left(\frac{N}{a}+1\right)-\frac{a+1}{a}. \end{array}$$

With the deductions above, Theorem 2 is proved. □

### 4.2. Temporary Gaps Only

#### 4.2.1. Linked List Arrangement

The proof of Theorem 3 is easily derivable from Equation (18), with the substitution of $n=\frac{N}{a}$:

(25) $$A\left(N,a\right)=\frac{N}{a}+\frac{a-1}{a}\approx n+1=C.\;□$$

The proofs of node heavy cases, Theorem 4, are the following.

According to arrangement *a)* we can write that n=N/a, and

(26) $$\begin{array}{cc} A(N,a) & =\sum_{i=1}^{n}\frac{2i-1}{N}+2n\frac{N-n}{N}\\ \; & =N[\frac{2a-1}{a^{2}}]=n\frac{2a-1}{a}. \end{array}$$

It is visible from the results that as *N* and *a* grow along with n=const. the value tends to 2n, which is not surprising considering that accumulation is possible merely in the last element.

Considering arrangement *b)* we will get that

(27) $$\begin{array}{cc} A(N,a) & =\sum_{i=1}^{n}\frac{2i-1}{N}+\frac{n+1}{2}\frac{N-n}{N}\\ \; & =\frac{1}{2}\frac{N+a-1}{a}. \end{array}$$

Comparing the result to Equation (25) we can see that the average SEARCH cost is half of that of the uniformly distributed one.

Arrangement *c)* can be expressed by the following equation:

(28) $$\begin{array}{cc} A(N,a) & =2\frac{N-n}{N}\sum_{i=1}^{n}\frac{2i-1}{N}\\ \; & =2\frac{N-n}{N}+\frac{2}{N}\sum_{i=1}^{n}i-\frac{\left(n-1\right)}{N}\\ \; & =2\frac{N-n}{N}+\frac{2}{N}\left(\frac{n(n+1)}{2}\right)-\frac{\left(n-1\right)}{N}\\ \; & =2\frac{N-n}{N}+\frac{n(n+1)}{N}-\frac{\left(n-1\right)}{N} \end{array}$$

The result shows that by increasing *N*, next an n=const., A(N,a) tends to 2:

(29) $$lim_{N\to \infty }A\left(N,a\right)=2.$$

The proof of Theorem 4 is completed.  □

#### 4.2.2. Completely Balanced Arrangement

The Theorem 5 can be easily proven based on Equation (6b):

(30) $$A\left(N,a\right)\frac{3}{2}log_{2}\left(\frac{N}{a}+1\right)+\frac{3a}{2N}log_{2}\left(\frac{N}{a}+1\right)-\frac{a+1}{a}$$

By replacing $\frac{N}{a}$ with *n*, and considering that during the examinations n=const.:

(31) $$\begin{array}{cc} A(n=const.,a) & =\frac{3}{2}log_{2}\left(n+1\right)+\frac{3}{2n}log_{2}\left(n+1\right)-\frac{a+1}{a}\\ \; & =c_{1}+c_{2}-\frac{a+1}{a}. \end{array}$$

As a>>0 (since *a* is increasing infinitely) we get the following approximation:

(32) A(N,a)≈C(n)−1.□

During the proofs above, we restricted our examinations to such cases where the requested key has already stored in the data structure. Of course the missing keys would modify the results: the interval length of the gaps should be considered, instead of the interval length of the nodes. Here, the weight of the half open intervals should be handled by care.

## 5. Evaluations and Simulations

Above, we analyzed the average time complexity of search operation of IMBT considering various input patterns. In the following, to put the IMBT in context, first we compare the cost of the search with the case of using other data structures in wide use. Throughout the comparisons, we consider the effect of permanent gaps and temporary gaps which, as we shall see, play a role only in the case of IMBT.

Then, we present the results of the selected simulations. In this case, we can point out hidden gains caused by the reduced number of nodes. These reduce, for instance, the cost of a tree rotation.

### 5.1. Evaluations

We compare IMBT with AVL balanced BST, hash tables, and Splay trees [19]. AVL was selected because AVL-based balancing has been applied by us for IMBT as well in our simulations. Hash was selected since its average time complexity of search operation is a constant time. Splay tree was chosen because it has a self-balancing structure influenced by the access pattern.

#### 5.1.1. Permanent Gaps

Table 1 compares the performance of IMBT (balanced—IMBT-B, linked list degenerated—IMBT-LLD) with data structures, in case there are permanent gaps.

As we can see from the table, the permanent gaps reduce the IMBT-LLD to a linked list with reduced number of nodes. The only gain here might be the constant divisor factor. In case *a* is high enough, the space complexity is a fraction of all other data structures.

Not surprisingly, IMBT-LLD has the worst performance on search time of all the data structures. For IMBT-B even the worst case has O(log(N)) time complexity, while a gain can originate from the reduced space complexity which can be significant, depending on the value of *a*.

#### 5.1.2. Temporary Gaps Only

Table 2 compares the performance of IMBT (balanced—IMBT-B, linked list degenerated—IMBT-LLD) with data structures, in case there are temporary gaps. From the three variants of IMBT-LLD, only the middle node-heavy variant will be covered.

The stochastically constant height of IMBT leads to stochastically constant search cost and constant space complexity.

Therefore, where it is possible, it is worth it to apply artificial maintenance insertion operations to eliminate the permanent gaps. Of course this is an application-specific decision, when, for instance, the user is aware that a reporting instrument failed, a range of keys surely would never arrive to the tree, otherwise the artificially inserted values override the real ones. Through a well-designed maintenance insertion the height of the tree probably can be kept under a certain limit.

### 5.2. Simulations

Below, some selected simulations are presented. A mixed statistical model was applied that was introduced in detail in [4]: The permanent gaps are induced by different Bernoulli distributions and temporary gaps are generated through distinct extents of shuffling with the help of distinct λ parameters of the exponential distribution.

During the simulations 1 million keys are always emitted, however, in case of permanent gaps/loss, a prefixed ratio/amount of the keys did not arrive to the trees.

During the simulations we investigated the evolution of the number of nodes, number of rotations, number of merges, etc., over the received keys and/or as a function of λ. In this contribution, we limit our attention to the number of nodes as a function of received keys and λ.

**Figure 12:**
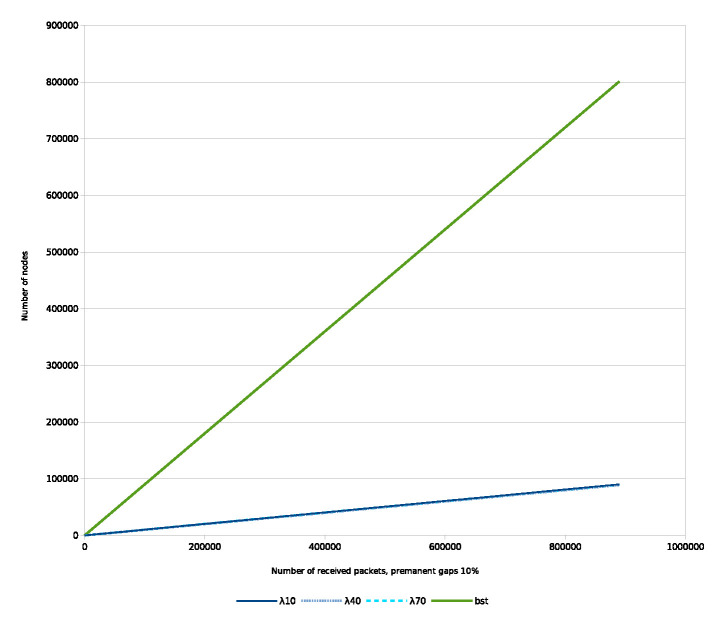
Time series of number of nodes for balanced BST and IMBT for permanent gaps.

We show the number of nodes over the received keys with different λ parameters, Figure 12. The ratio of the permanent loss is 10 percent.

In the case of BST, the required number of nodes is equal with the received number of keys.

Regarding IMBT, along with uniformly distributed gaps, the required number of nodes is proportional with N/a to store the keys which is practically independent from λ. It is a permanent space complexity gain compared to other data structures.

Taking into account the uniformly distributed character of the gaps and considering the Equations (6b) and (7), the IMBT provides time complexity advantage on average up to $N=a^{3}$ keys. Over that, only the space complexity gain remains. Otherwise, from a time complexity point of view IMBT behaves just like other BSTs.

**Figure 13:**
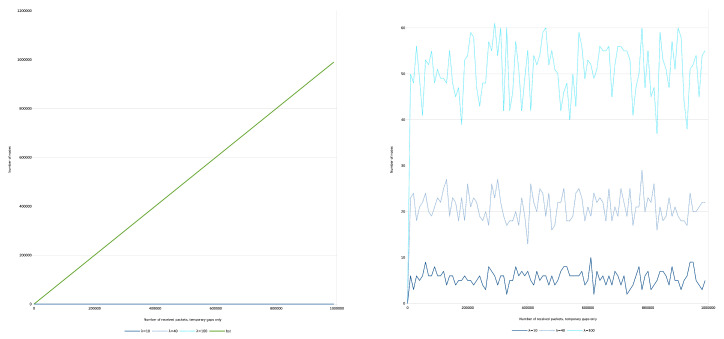
Number of nodes for balanced BST and IMBT for temporary gaps, as a function of the received keys/degree of shuffling.

Next, the number of nodes of the balanced BST and IMBT over the received keys in the case of temporary gaps only is evaluated, Figure 13.

In the case of BST, the result is similar to the previous one, which is visible on the left side of the figure.

However, in case of IMBT the situation is different. As we theoretically predicted the number of nodes becomes a stationary process in case of IMBT. This is visible on the right side of the figure, which are the magnified results of the bottom of left measurements.

That is, the simulation confirms Equation (32), according to which A(N,a) can be considered (stochastically) constant, that is the time complexity of the search operation is O(1). Moreover, the space complexity can also be considered constant, as stated above. Therefore, the IMBT can be a very efficient data structure both in terms of space/time complexity of the search operation.

**Figure 14:**
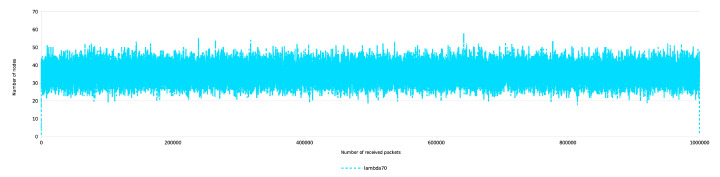
Balanced IMBT, temporary gaps only. Instantaneous number of nodes over received keys.

During the previous simulations only every 10,000th states were displayed. In the following, for demonstrating purposes, we provide the diagram, where all 1 million states are displayed with λ=70. From Figure 14 the stability of the process is clearly visible.

## 6. Conclusions

In the contribution above we have mixed together the Markov property based node number behavior and the corresponding contingency tables. In numerous cases, where it was possible, we gave a two-parameters-based formula to be able to estimate the average cost of search operations. To conclude, in this contribution, two distinct ways have been presented that enable the analysis, in the possession of a-priori knowledge on the traffic pattern, of the performance of the IMBT data structure, and a subsequent decision whether to apply it over another well known data structure, for the particular case.

## Figures and Tables

**Table 1:** Evaluation of time and space complexity of data structures in case of permanent gaps.

	Number of Keys	Number of Nodes	Space Complexity	Search Operation Time Complexity (Average)	Search Operation Time Complexity (Worst Case)
AVL Balanced BST	*N*	n=N	O(N)	O(log(N))	O(log(N))
Splay tree	*N*	n=N	O(N)	O(log(N))	O(log(N))
Hash	*N*	n=N	O(N)	O(1)	O(N)
IMBT-LLD	*N*	$n=\frac{N}{a}$	$O\left(\frac{2}{a}N\right)=O\left(N\right)$	$O\left(\frac{N}{a}+\frac{a-1}{a}\right)=O\left(N\right)$	$O\left(\frac{2}{a}N\right)=O\left(N\right)$
IMBT-B	*N*	$n=\frac{N}{a}$	$O\left(\frac{2}{a}N\right)=O\left(N\right)$	$O\left(\frac{3}{2}\log_{2}\left(\frac{N}{a}\right)\right)=O\left(log\left(N\right)\right)$	$O\left(2\times \log_{2}\left(\frac{N}{a}\right)\right)=O\left(log\left(N\right)\right)$

**Table 2:** Evaluation of time and space complexity of data structures in case of temporary gaps.

	Number of Keys	Number of Nodes	Space Complexity	Search Operation Time Complexity (Average)	Search Operation Time Complexity (Worst Case)
AVL Balanced BST	*N*	n=N	O(N)	O(log(N))	O(log(N))
Splay tree	*N*	n=N	O(N)	O(log(N))	O(log(N))
Hash	*N*	n=N	O(N)	O(1)	O(N)
Middle node-heavy IMBT-LLD	*N*	n=const.	O(2n=const.) = O(1)	O(n=const.) = O(1)	O(2n=const.) = O(1)
IMBT-B	*N*	n=const.	O(2n=const.) = O(1)	O(C(n=const.)−1) = O(1)	O(C(n=const.)−1) = O(1)

## References

[1] Cormen T.H., Leiserson C.E., Rivest R.L., Stein C. (2009). Introduction to Algorithms.

[2] Stoica I., Morris R., Liben-Nowell D., Karger D.R., Kaashoek M.F., Dabek F., Balakrishnan H. (2003). Chord: A Scalable Peer-to-peer Lookup Protocol for Internet Applications. IEEE/ACM Trans. Netw..

[3] Finta I., Farkas L., Sergyán S., Szénási S. Interval Merging Binary Tree. Proceedings of the ICA3PP 2017.

[4] Finta I., Élias G., Illés J. Packet Loss and Duplication Handling in Stream Processing Environment. Proceedings of the CINTI 2018.

[5] STORM—A Distributed Real-Time Computation System. http://storm.apache.org/documentation/Home.html.

[6] Finta I., Szénási S. (2019). State-space Analysis of the Interval Merging Binary Tree. Acta Polytech. Hung..

[7] Adelson-Velsky G., Landis E. (1962). An algorithm for the organization of information. Doklady Akademii Nauk.

[8] Bayer R. (1972). Symmetric binary B-Trees: Data structure and maintenance algorithms. Acta Inform..

[9] Lauritzen S.L. Lectures on Contingency Tables, 2002, Electronic edition, Aalborg University. http://www.stats.ox.ac.uk/~steffen/papers/cont.pdf.

[10] Barvionk A. (2008). Enumerating Contingency Tables via Random Permanents. Comb. Probab. Comput..

[11] Barvinok A., Luria A., Samorodnitsky A., Yong A. (2010). An approximation algorithm for counting contingency tables. Random Struct. Algorithms.

[12] Chung K.L. (1960). Markov Chains with Stationary Transition Probabilities.

[13] Meyn S.P., Tweedie R.L. (2012). Markov Chains and Stochastic Stability.

[14] Bernstein S.N. (1946). Theory of Probabilities.

[15] Doukhan P., Louhichi S. (1999). A new weak dependence condition and applications to moment inequalities. Stoch. Process. Their Appl..

[16] Lawder J., King P. (2000). Querying Multi-dimensional Data Indexed Using Hilbert Space-Filling Curve. ACM Sigmod Rec..

[17] Bóna M. (2002). A Walk Through Combinatorics: An Introduction to Enumeration and Graph Theory.

[18] Hardy G.H., Ramanujan S. (1918). Asymptotic Equatione in Combinatory Analysis. Proc. Lond. Math. Soc..

[19] Sleator D.D., Tarjan R.E. (1985). Self-Adjusting Binary Search Trees. J. ACM.

